# *SORL1* deficiency in human excitatory neurons causes APP-dependent defects in the endolysosome-autophagy network

**DOI:** 10.1016/j.celrep.2021.109259

**Published:** 2021-06-15

**Authors:** Christy Hung, Eleanor Tuck, Victoria Stubbs, Sven J. van der Lee, Cora Aalfs, Resie van Spaendonk, Philip Scheltens, John Hardy, Henne Holstege, Frederick J. Livesey

**Affiliations:** 1 UCL Great Ormond Street Institute of Child Health, Zayed Centre for Research into Rare Disease in Children, 20 Guilford Street, London WC1N 1DZ, UK; 2 Gurdon Institute, University of Cambridge, Cambridge CB2 1QN, UK; 3 Alzheimer Center Amsterdam, Department of Neurology, Amsterdam Neuroscience, Vrije Universiteit Amsterdam, Amsterdam UMC, Amsterdam, the Netherlands; 4 Department of Clinical Genetics, Amsterdam UMC, Amsterdam, the Netherlands; 5 Delft Bioinformatics Lab, Delft University of Technology, Delft, the Netherlands; 6 UK Dementia Research Institute and Department of Neurodegenerative Disease and Reta Lila Weston Institute, UCL Queen Square Institute of Neurology and UCL Movement Disorders Centre, University College London, London, UK; 7 Institute for Advanced Study, The Hong Kong University of Science and Technology, Hong Kong, China

**Keywords:** endosome, lysosome, autophagy, Alzheimer's disease, amyloid precursor protein, SORL1, iPSC

## Abstract

Dysfunction of the endolysosomal-autophagy network is emerging as an important pathogenic process in Alzheimer’s disease. Mutations in the sorting receptor-encoding gene *SORL1* cause autosomal-dominant Alzheimer’s disease, and *SORL1* variants increase risk for late-onset AD. To understand the contribution of *SORL1* mutations to AD pathogenesis, we analyze the effects of a *SORL1* truncating mutation on SORL1 protein levels and endolysosome function in human neurons. We find that truncating mutation results in SORL1 haploinsufficiency and enlarged endosomes in human neurons. Analysis of isogenic *SORL1* wild-type, heterozygous, and homozygous null neurons demonstrates that, whereas *SORL1* haploinsufficiency results in endosome dysfunction, complete loss of *SORL1* leads to additional defects in lysosome function and autophagy. Neuronal endolysosomal dysfunction caused by loss of SORL1 is relieved by extracellular antisense oligonucleotide-mediated reduction of APP protein, demonstrating that PSEN1, APP, and SORL1 act in a common pathway regulating the endolysosome system, which becomes dysfunctional in AD.

## Highlights

• Alzheimer’s disease *SORL1* truncating mutations cause SORL1 haploinsufficiency
• Reduced SORL1 protein results in endosome defects in human neurons
• Complete loss of SORL1 protein causes endosome, lysosome, and autophagy defects
• Reduction of APP rescues endolysosome and autophagy defects in *SORL1*-null neurons

## Graphical abstract

**Figure:**
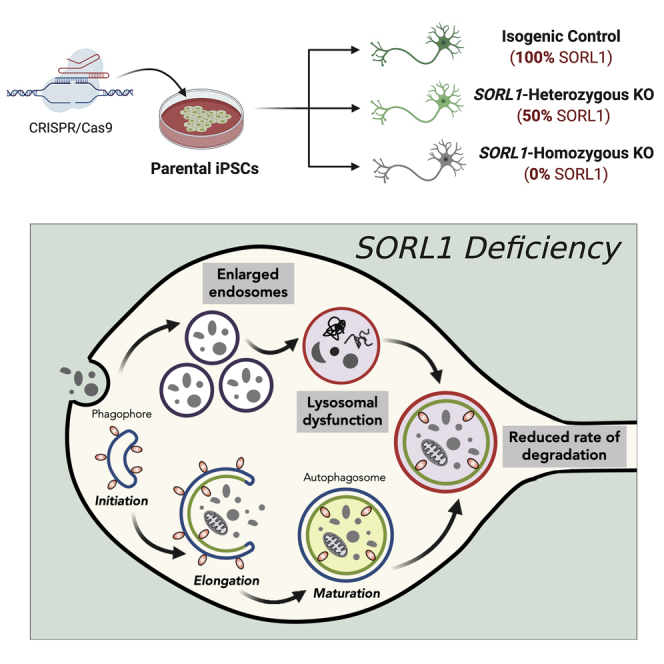

## Teaser

Hung et al. demonstrate that truncating mutations in *SORL1* result in endosome defects in human neurons. Complete loss of SORL1 causes additional defects in lysosome function and autophagy. Endolysosomal dysfunction due to *SORL1* loss of function is APP dependent, as it is relieved by antisense oligonucleotide-mediated reduction of APP protein.

## Introduction

Dysfunction of the endolysosome-autophagy network is an early pathological feature of Alzheimer’s disease (AD) (Nixon, 2017) and is emerging as an important pathogenic process in the monogenic, inherited form of the disease (Hung and Livesey, 2018; Kwart et al., 2019). However, the contribution of dysfunction in these systems to pathogenesis of the late-onset, sporadic form of the disease is less clear. Late-onset, sporadic AD is the most common form of dementia, and there are currently no effective treatments that modify disease progression (De Strooper and Karran, 2016). The recent identification in genome-wide association studies (GWASs) of variants associated with genes with roles in the endolysosomal and autophagy systems (Karch and Goate, 2015) suggest that the endolysosomal-autophagy system may act as a common primary target for disruption by diverse genetic risk factors for late-onset AD.

Mechanistic studies of the sortilin-related receptor 1 (*SORL1*) gene, which encodes a regulator of protein trafficking between the *trans*-Golgi network and endosomes, including amyloid precursor protein (APP) (Andersen et al., 2005; Mehmedbasic et al., 2015; Rogaeva et al., 2007; Young et al., 2015), provide an opportunity to analyze a pathogenic cellular pathway common to both monogenic and sporadic AD. Converging lines of evidence from studies in cell culture (Rogaeva et al., 2007), knockout (KO) mouse models (Caglayan et al., 2014; Dodson et al., 2008), and individuals with AD indicate that loss of SORL1 function (i.e., haploinsufficiency) is a cause of monogenic AD, in addition to autosomal dominant missense mutations in *APP*, *PSEN1*, and *PSEN2* (Sager et al., 2007; Scherzer et al., 2004). Notably, both stop-gain and frameshift mutations in *SORL1* are exclusively observed in AD patients (Holstege et al., 2017; Raghavan et al., 2018), providing direct genetic evidence that truncating variants of *SORL1* are highly penetrant. From ongoing GWASs, variants near and in the *SORL1* gene have been found to confer significantly elevated risk for developing the disease (Holstege et al., 2017; Raghavan et al., 2018; Rogaeva et al., 2007). Therefore, *SORL1* is potentially involved in the pathogenesis of both early- and late-onset AD (Bettens et al., 2008; Caglayan et al., 2012; Kimura et al., 2009; Nicolas et al., 2016; Rogaeva et al., 2007; Tan et al., 2009; Verheijen et al., 2016).

*SORL1* is highly expressed in the brain and acts as a sorting receptor for APP, regulating its trafficking between the endosome and the *trans*-Golgi network (Andersen et al., 2016). Trafficking of APP through the endosomal-lysosomal and autophagic compartments, where the various secretases that proteolytically process APP reside, is a regulatory step that determines the processing fates of APP (O’Brien and Wong, 2011). SORL1 promotes transport of APP from endosomes to the *trans*-Golgi network within the retromer complex and restricts its exit from the Golgi, thereby reducing amyloidogenic processing of APP that would generate Aβ peptides (Rogaeva et al., 2007). Thus, genetic deletion of *SORL1* in mouse models enhances Aβ production by increasing the delivery of APP to the endocytic compartments that favor amyloidogenic breakdown (Dodson et al., 2008). SORL1 also acts as a sorting factor for Aβ, directing it to the lysosome for degradation, further reducing the amyloidogenic burden (Caglayan et al., 2014).

We and others have shown that accumulation of APP protein fragments generated from the amyloidogenic pathway within endosomal compartments disrupts endolysosomal function and autophagy (Colacurcio et al., 2018; Jiang et al., 2016, 2019; Kwart et al., 2019; Lauritzen et al., 2016, 2019; Nixon, 2007, 2017; Nixon et al., 2005). Recent studies have shown that human cortical neurons derived from individuals with mutations in *APP* and *PSEN1*, which are causal for familial AD, lead to major defects in endolysosome function and autophagy (Hung and Livesey, 2018; Kwart et al., 2019). We report here that *SORL1* mutations lead to reduced levels of SORL1 protein in human neurons, and that loss of SORL1 protein results in neuronal endolysosome and autophagy defects. We find that SORL1 acts in the same pathway as APP and PSEN1 that regulates endolysosome function, because antisense oligonucleotide-mediated reduction of APP in *SORL1*-null neurons prevents defects in the endolysosome system.

## Results

### Enlarged endosomes in neurons generated from an individual with dementia as a result of a truncating *SORL1* mutation

**Figure 1:**
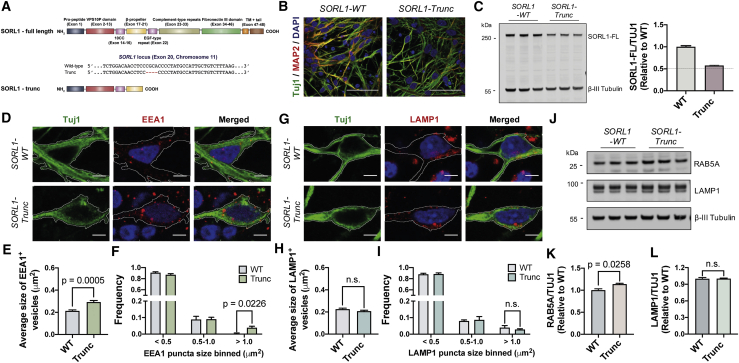
Enlarged endosomal phenotype in patient iPSC-derived neurons carrying *SORL1* truncation mutation (A) Schematic of the truncating *SORL1* mutation at exon 20 (SORL1-trunc) of the *SORL1* gene. (B) Representative immunohistochemistry of neurons from non-demented control or carrying *SORL1* mutation (green, β3-tubulin; red, MAP2 and blue, DAPI). Scale bars, 50 μm. (C) Total full-length SORL1 levels are reduced by approximately 50% in *SORL1* mutant neurons compared with non-demented control as detected by western blot analysis. Representative western blots of SORL1 and neuron-specific β3-tubulin in control and *SORL1* mutant neurons are shown. Levels of full-length SORL1 were calculated relative to those of non-demented controls (n = 3). (D) Representative immunohistochemistry of iPSC-derived neurons expressing EEA1 proteins (red, EEA1; green, β3-tubulin; blue, DAPI). Scale bars, 5 μm. (E and F) A significant increase in the average size of early endosomes (E) and frequency (F) of early endosomes with size > 1 μm^2^ in human cortical excitatory neurons with *SORL1* mutations compared with non-demented control (n = 18–34 neurons). (G) Representative immunohistochemistry of iPSC-derived neurons expressing LAMP1 proteins (red, LAMP1; green, β3-tubulin; blue, DAPI). Scale bars, 5 μm. (H and I) No significant changes in the average size of late endosomes/lysosomes (H) and frequency (I) of late endosomes/lysosomes in human cortical excitatory neurons with *SORL1* mutations compared with non-demented control (n = 16–17 neurons). (J–L) Total Rab5A levels are significantly increased in *SORL1* mutant neurons (90 days post-neural induction), but endogenous LAMP1 levels are not altered, as detected by western blot analysis. Representative western blots of Rab5A, LAMP1, and neuron-specific β3-tubulin in control and *SORL1* mutant neurons are shown (J). Levels of Rab5A (K) and LAMP1 (L) were calculated relative to β3-tubulin (n = 3).

To understand how *SORL1* truncating variants may impact the onset and progression of AD, we generated cortical excitatory neurons from induced pluripotent stem cells (iPSCs) derived from an individual clinically diagnosed with AD and carrying a truncating *SORL1* mutation at exon 20 (Figure 1A), who was also homozygous for the APOE4 allele, which independently increases the risk for AD by approximately 12-fold (Kim et al., 2009). Human iPSC-derived cortical excitatory (glutamatergic) neurons were generated using standard methods (Shi et al., 2012) and confirmed as being cortical in neuronal identity (Figure 1B). In neurons generated from the patient-derived iPSCs, full-length SORL1 protein levels were at ~50% of those detected in a non-demented control (Figure 1C). This indicated that the truncating mutation in this individual disrupted the reading frame, leading to SORL1 haploinsufficiency.

Within neurons generated from the *SORL1* mutant iPSCs, we measured the number and size of early endosomes by confocal imaging of endogenous EEA1 (Early Endosome Antigen 1), a well-established early endosome protein (Gorvel et al., 1991; Simonsen et al., 1998). We observed a significant increase in the average size of early endosomes (EEA1^+^ puncta) in *SORL1* mutant neurons compared with non-demented controls (Figures 1D and 1E). In addition, we observed a significant increase in the frequency of larger early endosomes (>1 μm^2^) in *SORL1* mutant neurons (Figure 1F).

To further investigate whether *SORL1* truncating variants impair the downstream events in the endocytic pathway, we measured the size and number of late endosomes/lysosomes by immunostaining for the endogenous lysosomal glycoprotein LAMP1 (Lysosomal-associated membrane protein 1). We found that there were no significant changes in the size and area occupied by LAMP1^+^ puncta in *SORL1* mutant neurons compared with non-demented controls (Figures 1G–1I). Consistent with these findings, the *SORL1* mutant neurons demonstrated a significant increase in total amounts of endosomal Rab5A protein, with no changes in the level of LAMP1 protein compared with the non-demented controls, as assessed by quantitative immunoblot (Figures 1J–1L). Thus, these findings indicate that human cortical neurons derived from individuals with dementia as a result of truncating *SORL1* mutations are haploinsufficient for SORL1 protein and have pronounced changes in endosomes, but no overt changes in lysosomes at this relatively early stage.

### Complete loss of SORL1 results in early endosome enlargement and lysosome dysfunction

**Figure 2:**
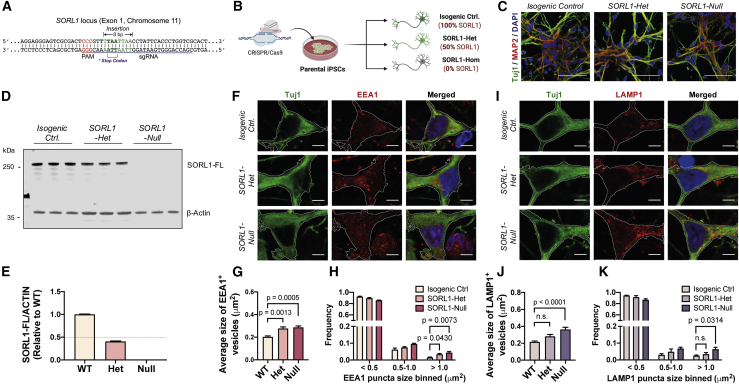
*SORL1* deficiency leads to early endosome enlargement and defective lysosomal function (A and B) Schematic of the CRISPR/Cas9 gene-mediated strategy used to generate an isogenic allelic series from the parental cell line. Created with BioRender. (C) Representative immunohistochemistry of neurons from isogenic control, heterozygous, and homozygous SORL1 KO human iPSCs (green, β3-tubulin; red, MAP2; blue, DAPI). Scale bars, 50 μm. (D and E) Total SORL1 levels are reduced by approximately 50% in *SORL1* heterozygous KO neurons and completely absent in *SORL1* homozygous KO neurons compared with isogenic control as detected by western blot analysis. Representative western blots of SORL1 and neuron-specific β3-tubulin are shown (D). Levels of SORL1 (E) were calculated relative to those of isogenic controls (n = 3). (F) Representative immunohistochemistry of iPSC-derived neurons expressing EEA1 proteins (red, EEA1; green, β3-tubulin; blue, DAPI). Scale bars, 5 μm. (G and H) A significant increase in the average size of early endosomes (G) and frequency (H) of early endosomes with size > 1 μm^2^ in both heterozygous and homozygous *SORL1* KO neurons compared with isogenic control (n = 16–32 neurons). (I) Representative immunohistochemistry of iPSC-derived neurons expressing LAMP1 proteins (red, LAMP1; green, β3-tubulin; blue, DAPI). Scale bars, 5 μm. (J and K) A significant increase in the average size (J) of late endosomes/lysosomes and frequency (K) of late endosomes/lysosomes with size > 1 μm^2^ in *SORL1*-null neurons compared with isogenic control (n = 17–29 neurons).

To further interrogate the function of SORL1 in the endolysosomal system in human neurons, we used CRISPR-Cas9-mediated homologous recombination targeting exon 1 of the *SORL1* gene to generate an isogenic null allelic series (see Figure 2A and STAR Methods for details of the targeting strategy). In addition, these were all generated from a parental cell line (KOLF2, which was derived by the Human Induced Pluripotent Stem Cell Initiative [HipSci] consortium) (Bruntraeger et al., 2019; Kilpinen et al., 2017) that was APOE3/3, to distinguish the contribution of *SORL1* from that of the *APOE4* allele. The isogenic series is represented by isogenic control (isogenic ctrl), heterozygous (*SORL1*-het), and homozygous (*SORL1*-null) *SORL1* KO human iPSCs (Figures 2A and 2B). As expected, heterozygous and homozygous *SORL1* KOs led to a ~50% loss and complete absence of full-length SORL1 protein, respectively, as assessed by western blotting, compared with the isogenic parental iPSC line (Figures 2C–2E).

In heterozygous and homozygous *SORL1*-null neurons compared with isogenic controls, all generated from the isogenic allelic series of the *SORL1* KO iPSCs, we detected a significant increase in the average size of early endosomes (EEA1^+^ puncta) (Figures 2F and 2G). In addition, we observed an increase in the frequency of larger early endosomes (>1 μm^2^) in heterozygous and homozygous *SORL1*-null neurons (Figure 2H). Our results are consistent with the findings in the patient-derived *SORL1* heterozygous mutant neurons and further confirmed that deficiency of SORL1 protein leads to enlarged early endosomes in human neurons.

As described above, loss of function of one allele of *SORL1* in patient-derived neurons was associated with endosome defects, without additional lysosome or autophagy phenotypes. We hypothesized that a complete depletion of SORL1 protein (homozygous *SORL1*-null) in human neurons may result in a more severe phenotype compared with the heterozygous KO and further impair downstream events in the endocytic pathway. To explore this further, we measured late endosome/lysosome size and number by confocal microscopy (Figures 2I–2K). We observed a significant increase in the average size of late endosomes/lysosomes (LAMP1^+^ puncta) (Figures 2I and 2J) and in the proportion of larger lysosomal LAMP1^+^ puncta (>1 μm^2^) in homozygous *SORL1*-null neurons compared with isogenic controls, but not in heterozygous *SORL1*-null neurons (Figure 2K). Therefore, our results indicate that complete loss of *SORL1* leads to major defects in endosomal and lysosomal function, in contrast with heterozygous mutations that primarily cause endosome defects at this early stage.

### Complete loss of SORL1 alters APP processing and increases Aβ production in iPSC-derived cortical neurons

**Figure 3:**
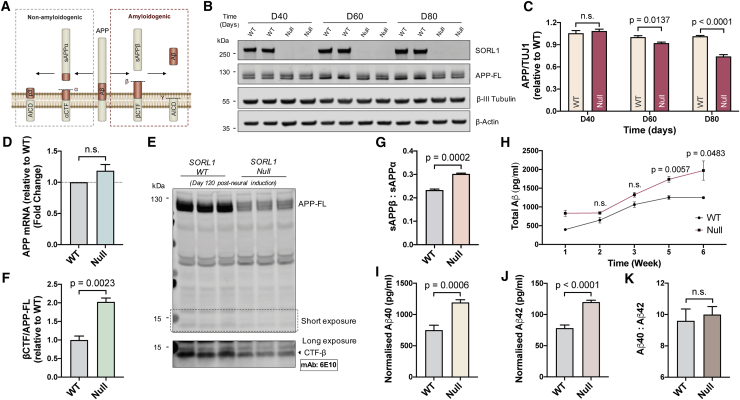
*SORL1* depletion in human iPSC-derived neurons increases Aβ production (A) Schematic of APP processing pathways. (B and C) Representative western blots of full-length SORL1, APP, neuron-specific β3-tubulin, and β-actin in isogenic control and *SORL1*-null neurons are shown (B). Levels of APP (C) were calculated relative to those of isogenic control (n = 4). (D) Quantification of *APP* mRNA levels by qRT-PCR in isogenic control and *SORL1*-null neurons. Values are relative to those of isogenic control (n = 3). (E and F) Representative western blot of full-length APP in isogenic control and *SORL1*-null neurons (120 days post-neural induction) is shown (E). Ratios of βCTF/APP-FL (F) were calculated relative to those of isogenic control (n = 3). (G) Quantification of sAPPα/sAPPβ ratio in isogenic control and *SORL1*-null neurons (n = 3). (H) *SORL1*-null neurons have a significant increase in production of total extracellular Aβ peptides over 6 weeks (post-neural induction) in culture compared with isogenic control neurons (n = 3). (I–K) At 6 weeks in culture, *SORL1*-null neurons exhibit a significant increase in production of both Aβ40 peptides (I) and Aβ42 peptides (J), with no change in their relative amounts (K) (n = 6).

Given previous reports of SORL1’s function as a neuronal sorting receptor for APP (Andersen et al., 2005; Rogaeva et al., 2007), we studied the effect of complete loss of SORL1 on APP processing in human iPSC-derived cortical neurons (Figure 3A). By quantitative immunoblotting, we observed that the levels of full-length APP protein decreased over time in *SORL1*-null neurons compared with isogenic ctrls (Figures 3B and 3C). No difference in *APP* mRNA expression between *SORL1*-null neurons and isogenic controls was detected by qRT-PCR (Figure 3D), indicating that the decrease in APP protein in *SORL1*-null neurons is post-transcriptional and most likely a result of altered APP proteostasis.

To further understand how complete absence of SORL1 in human neurons alters processing of APP (Figure 3A), we measured the relative amounts of different APP-derived peptides and proteins generated from the amyloidogenic pathway (Figures 3E–3K). There was a significant increase in the ratio of the beta-secretase-generated C-terminal fragment of APP (detected by 6E10 antibody) relative to full-length APP (βCTF/APP-FL) in *SORL1*-null neurons compared with the isogenic controls (Figures 3E and 3F). This was accompanied by a significant increase in the ratio of extracellular soluble APPβ (sAPPβ) to sAPPα (Figure 3G), indicating a relative shift toward β-secretase-mediated Aβ peptide production. This was confirmed by analysis of production of extracellular Aβ peptides over the course of 6 weeks in culture, which found a significantly increased production of Aβ peptides by *SORL1*-null neurons compared with the isogenic controls over time (Figure 3H), which included both Aβ40 and Aβ42 peptides (Figures 3I and 3J), with no change in their relative amounts (Figure 3K). Together, our findings suggest that absence of SORL1 in human neurons alters the processing of APP by shifting to the amyloidogenic pathway, resulting in increased APP proteolysis and production of all Aβ peptides.

### Loss of SORL1 increases APP/BACE1 interaction in late endosomes/lysosomes and perturbs autophagy in human neurons

**Figure 4:**
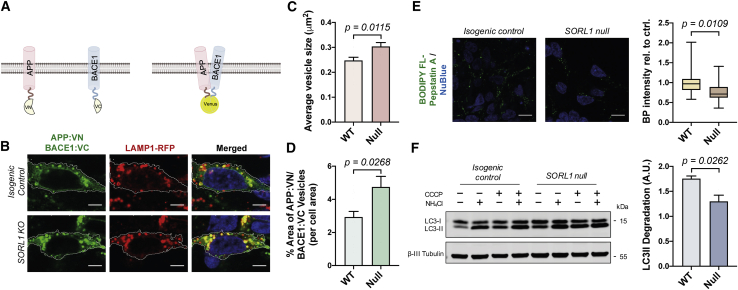
*SORL1* knockout enhances APP/BACE1 interactions and reduces autophagy flux (A) Schematic of the assay used to detect APP and BACE1 interactions. APP and BACE1 were tagged to complementary VN and VC fragment of Venus protein, respectively. Interaction of APP and BACE1 leads to the reconstitution of Venus fluorescence. Created with BioRender. (B) Representative images of neurons expressing APP:VN, BACE1:VC, and LAMP1:RFP (red, LAMP1; green, APP:VN/BACE1:VC; blue, nuclei labeled with NucBlue). Scale bars, 5 μm. (C and D) A significant increase in the average size of Venus-positive puncta (C) and % area of Venus-positive puncta (D) in *SORL1*-null neurons compared with isogenic control (n = 14–21 neurons). (E) Representative images and quantification of BODIPY FL-Pepstatin A (BP) labeling in isogenic control and *SORL1*-null neurons (n = 18–20 neurons). Scale bars, 10 μm. (F) Autophagosome degradation was significantly reduced in *SORL1* KO neurons compared with isogenic control, as calculated from the western blot analysis. Representative western blots of LC3I/II and neuron-specific β3-tubulin from neurons derived from *SORL1*-null and isogenic iPSCs are shown. Autophagosome degradation following autophagy induction with CCCP (20 μM) in the absence or presence of NH_4_Cl was calculated from three independent experiments (n = 3).

To investigate whether the loss of SORL1 function increases interactions between APP and BACE1 in endosomes and lysosomes, which would favor amyloidogenic processing, we applied a well-characterized APP/BACE1 Venus fluorescence complementation assay to study the interaction between APP and BACE1 (Das et al., 2016) (Figure 4A). In this assay, direct interaction of APP fused to the N terminus of Venus (APP:VN) with a BACE1-C-terminal Venus protein (BACE1:VC) leads to the reconstitution of Venus fluorescence (Das et al., 2016). In both isogenic control and *SORL1*-null neurons, we observed the majority of APP:VN/BACE1:VC-positive vesicles residing in late endosomes/lysosomes (as indicated by colocalization with LAMP1-RFP-labeled vesicles) (Figure 4B). However, there was a significant increase in the size of Venus^+^ vesicles within *SORL1*-null neurons and the area of the neuron occupied by those vesicles (Figures 4C and 4D), consistent with an increase in APP/BACE1 interactions in *SORL1*-null neurons compared with the isogenic controls.

Given that altered APP processing caused by *APP* and *PSEN1* mutations leads to defects in lysosomal function and the degradative phase of autophagy, we measured activity of lysosomal hydrolases and autophagic flux (Rubinsztein et al., 2009) in isogenic control and *SORL1*-null neurons (Figures 4E and 4F). We first measured the level of activation of cathepsin D (CTSD), a major lysosomal aspartyl protease, using BODIPY FL-Pepstatin A (BP), which is an affinity reagent that binds to the enzymatic active form of CTSD (Chen et al., 2000; Jiang et al., 2019). We observed a significant reduction in the intensity of BP fluorescence in *SORL1*-null neurons (Figure 4E), indicating a reduction in the level of lysosomal CTSD activity in *SORL1*-null neurons compared with the isogenic controls.

We also measured autophagic flux using a previously reported quantitative approach (Rubinsztein et al., 2009). In this assay, autophagy was induced in both isogenic control and *SORL1*-null neurons by mitochondrial respiratory chain uncoupler (CCCP) treatment, confirmed by significant upregulation of autophagosome LC3-II (Figure 4F). To distinguish between the synthetic and degradative phases of autophagy, we blocked autophagic degradation by addition of ammonium chloride (NH_4_Cl) in the presence of CCCP and compared LC3-II levels between neurons treated with CCCP to induce autophagy and those treated with CCCP and NH_4_Cl (blocking autophagosome degradation). Under those conditions, the elevation of LC3-II levels was significantly higher in isogenic control neurons than in *SORL1*-null neurons (Figure 4F), indicating that loss of SORL1 impairs autophagosome degradation.

### Extracellular administration of *APP*antisense oligonucleotides rescues endolysosome and autophagy dysfunction in *SORL1*-null neurons

**Figure 5:**
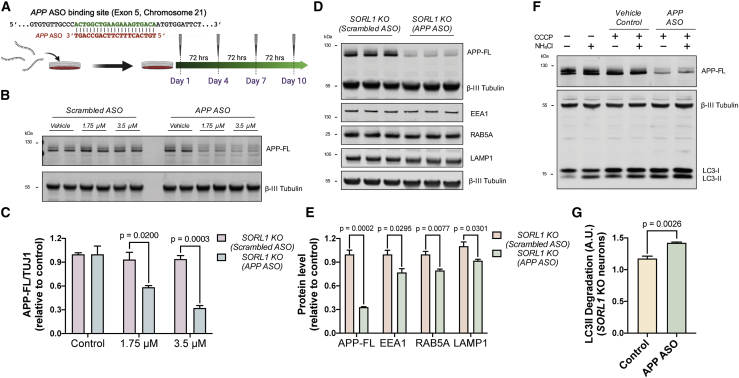
*APP* antisense oligonucleotides rescue endolysosomal dysfunction in *SORL1* KO neurons (A) Schematic of the binding region of *APP* antisense oligonucleotides and the experimental design; neurons were treated from day 55 to day 65 post-induction with *APP* antisense oligonucleotides. Created with BioRender. (B and C) *SORL1* KO neurons treated with *APP* antisense oligonucleotides for a 10-day period exhibit a dose-dependent decrease in APP protein. Representative western blots of APP and neuron-specific β3-tubulin in *SORL1* KO neurons treated with or without *APP* antisense oligonucleotides are shown in (B). Levels of APP-FL (C) were calculated relative to β3-tubulin (n = 3). (D and E) Total APP-FL, EEA1, Rab5A, and LAMP1 levels are significantly reduced in *SORL1* KO neurons treated with *APP* antisense oligonucleotides, as detected by western blot analysis. Representative western blots of APP, EEA1, Rab5A, LAMP1, and neuron-specific β3-tubulin are shown in (D). Levels of APP, EEA1, Rab5A, and LAMP1 (E) were calculated relative to β3-tubulin (n = 3). (F and G) Autophagosome degradation was significantly increased in *SORL1* KO neurons treated with APP antisense oligonucleotides, as calculated from the western blot analysis. Representative western blots of LC3I/II and neuron-specific β3-tubulin are shown in (F). (G) Autophagosome degradation following autophagy induction with CCCP (20 μM) in the absence or presence of NH4Cl in *SORL1* KO neurons treated with or without *APP* antisense oligonucleotides was calculated from three independent experiments (n = 3).

The data reported above demonstrate that loss of SORL1 function enhances the net delivery of APP to endocytic compartments, increasing beta- and gamma-secretase processing of APP, and that SORL1 loss of function leads to endolysosome and autophagy defects in human neurons. To formally test the dependency of the endolysosome and autophagy defects on APP, we applied extracellular antisense oligonucleotides to reduce neuronal APP protein levels in *SORL1*-null neurons (Figure 5A).

To do so, we used an antisense oligonucleotide (ASO) consisting of a central gap region of 10 2′-deoxynucleotides flanked on both sides by five 2′-methoxyethyl nucleotides wings that has been previously shown to effectively target the *APP* mRNA reducing APP protein levels (Dobie, 2003) (Figure 5A). ASOs were delivered to *SORL1*-null neurons by addition to extracellular media for a 10-day period, which resulted in a dose-dependent decrease in APP protein, with the highest concentration (3.5 μM) reducing APP levels to less than 30% of control levels (Figures 5B and 5C).

A 10-day treatment of *SORL1*-null neurons with APP ASOs resulted in a significant decrease in total EEA1, Rab5a, and LAMP1 protein levels in *SORL1*-null neurons (Figures 5D and 5E), indicating that reduction of APP protein rescues endolysosomal dysfunction in *SORL1*-null neurons. To test whether the reduction in APP protein in *SORL1*-null neurons also relieved autophagy dysfunction, we applied the quantitative autophagic flux assay described above (Figures 5F and 5G). As with endolysosome protein levels, we found that APP ASO treatment rescued autophagy defects, significantly increasing the rate of autophagosome degradation in particular (Figure 5G), although not to the levels measured in control neurons (Figure 4F).

## Discussion

We report here that human cortical neurons derived from an individual with dementia as a result of a heterozygous *SORL1* truncation mutation have half of control levels of SORL1 protein and disrupted endosomal trafficking, as do *SORL1* heterozygous null neurons. Generating neurons completely null for *SORL1* resulted in more severe phenotypes, including lysosome dysfunction and defects in the degradative phase of autophagy. Importantly, we observed that these endolysosomal and autophagy defects are dependent on the APP protein. In addition, we find that extracellular ASOs are an effective approach to reduce APP protein levels in human neurons, indicating that this therapeutic approach has considerable potential in monogenic AD due to *SORL1*, *APP*, or *PSEN1* mutations.

We and others recently reported that human cortical neurons carrying mutations in *APP* and *PSEN1*, which are causal for familial AD, lead to major defects in endolysosomal function and autophagy (Kwart et al., 2019). The findings reported here indicate that *PSEN1*, *APP*, and *SORL1* act in a common pathway that primarily regulates endosome function: heterozygous mutations in *SORL1* and *PSEN1* lead to defects in endosome function, whereas homozygous mutations in *SORL1* lead to defects in endosome, lysosome, and autophagosome function. Furthermore, APP has a central role in mediating the function of this pathway, given that reducing or knocking out APP rescues *SORL1*- and *PSEN1*-mediated phenotypes (Hung and Livesey, 2018), respectively. The specific endosomal process mediated by APP is currently not clear but has features of a regulated trafficking event that is licensed or regulated by APP.

However, it is not clear if and how the SORL1-APP interaction is related to APP’s processing by beta- and gamma-secretase, because it has been shown that small-molecule inhibition of BACE1 does not prevent endosome defects in *SORL1* null neurons (Knupp et al., 2020). In addition, APP may not be the only SORL1 cargo that contributes to *SORL1* KO neuronal endolysosome and autophagy dysfunction, because SORL1 regulates the transport of many different proteins (Fjorback et al., 2012; Glerup et al., 2013; Klinger et al., 2011). We find here that ASOs that reduce APP to less than 30% of wild-type levels do not completely rescue autophagy dysfunction; thus, the incomplete autophagy rescue may be due to the remaining APP protein or by the contribution of other SORL1 cargoes.

The data reported here suggest either reducing APP or increasing SORL1 function as potential therapeutic strategies in monogenic AD. SORL1 is a unique sorting receptor for directing APP to a non-amyloidogenic pathway, and the brain concentrations of SORL1 are inversely correlated with Aβ levels in mouse models and AD patients, suggesting that increasing expression of SORL1 receptor could be a novel therapeutic strategy for reducing the amount of amyloidogenic products. Overexpression of SORL1 reduces Aβ generation by altering the trafficking route of APP without affecting the processing of other BACE1 and γ-secretase substrates (Caglayan et al., 2014). Therefore, this strategy could potentially prevent the side effects associated with the use of beta- or gamma-secretase inhibitors.

The familial and sporadic forms of AD are clinically and pathologically similar, suggesting a convergence point in their pathological sequence. However, the common pathogenic pathways shared by both forms of the disease have remained unclear. Our data suggested that dysfunction of the endolysosomal-autophagic system represents a convergent mechanism shared by familial and sporadic forms of AD. As such, it opens up avenues to explore whether correcting defects in these systems could potentially alter progression of both forms of the disease. This is important because GWASs clearly demonstrate the multifactorial complexity of AD pathogenesis, highlighting the need to understand and elucidate the cellular mechanisms involved and how they converge, leading to the decline in neuronal function.

## STAR★Methods

### Key resources table

**Table:** 

REAGENT or RESOURCE	SOURCE	IDENTIFIER
**Antibodies**		

Mouse anti-β-Amyloid, 1-16	BioLegend	Cat#803001; RRID: AB_2564653
Mouse monoclonal anti-APP C-Terminal Fragment	BioLegend	Cat#802801; RRID: AB_2564648
Rabbit polyclonal anti-Tubulin β-3	BioLegend	Cat#802001; RRID: AB_2564645
Mouse monoclonal anti-Tubulin β-3	BioLegend	Cat#801201; RRID: AB_2313773
Mouse monoclonal anti-β-actin	Sigma	Cat#A2228; RRID: AB_476697
Rabbit polyclonal anti-LAMP1	abcam	Cat#Ab62562 RRID: AB_2134489
Rabbit polyclonal anti-LC3B	Sigma	Cat#L7543; RRID: AB_796155
Chicken polyclonal anti-MAP2	abcam	Cat#Ab5392; RRID: AB_2138153
Rabbit monoclonal anti-EEA1	abcam	Cat#Ab109110; RRID: AB_10863524
Rabbit monoclonal anti-SORL1	abcam	Cat#Ab190684

**Oligonucleotides**		

gRNA: CGACCAGGGTGAATAGGAAC	This paper	N/A
*APP* ASO: TGTCACTTTCTTCAGCCAGT	This paper	N/A
*APP* qRT-PCR Forward Primer: AAAACGAAGTTGAGCCTG	This paper	N/A
*APP* qRT-PCR Reverse Primer: CCGTCTTGATATTTGTCAACC	This paper	N/A

**Software and algorithms**		

ImageJ/FIJI	https://imagej.net/Welcome	RRID: SCR_003070
GraphPad Prism	https://www.graphpad.com/	RRID: SCR_002798

### Resource availability

#### Lead contact

Further information and requests for resources and reagents should be directed to and will be fulfilled by the Lead Contact, Rick Livesey (r.livesey@ucl.ac.uk).

#### Materials availability

Cell lines generated in this study may be available from the Lead Contact with a completed Materials Transfer Agreement. Restrictions may apply to the availability of the cell lines due to our need to maintain the stock.

#### Data and code availability

No datasets were generated during this study.

### Experimental model and subject details

Non-demented control iPSC lines: Non-Demented-Control (NDC) (Israel et al., 2012), was previously reported and characterized. SORL1 mutant fibroblasts were obtained from the Alzheimer Dementia Cohort from the Amsterdam Alzheimer Center, and previously described (Holstege et al., 2017). All patients signed informed consent for skin biopsies for the generation of iPSCs. This research was carried out in accordance with the UK Code of Practice for the Use of Human Stem Cell Lines.

The specific mutation studied here was: SORL1 (NM_003105.5):c.2882_2885del, p.(His962Profs^∗^45); the fibroblast cell line was given the in-house reference Cell line 666: SORL1-trunc.

### Method details

#### Generation and characterization of patient-specific iPSCs

Fibroblasts were reprogrammed into iPSCs using non-integrating, Sendai virus carrying the reprogramming factors Oct4, Sox2, Klf4, and c-Myc (CytoTune, ThermoFisher). Briefly, fibroblasts were transduced with viral particles, plated at low densities and maintained in iPSC media until colonies with clear iPSC morphology were observed. Colonies with iPSC morphology were then picked manually and expanded. Total RNA was extracted from iPSC lines after 10 passages and analyzed with the NanoString nCounter system using a pre-designed codeset, which contains probes for detection of Sendai viral transgenes, pluripotency, Mycoplasma species and housekeeping genes. Gene expression levels were analyzed using the nSolver Analysis Software (NanoString) and results were compared with a Sendai-positive and a Sendai-negative sample to ensure the iPSC lines were free from reprogramming virus.

#### Generation of isogenic heterozygous and homozygous SORL1 knockout iPSCs

Optimal gRNA design was performed using the Integrated DNA Technologies (IDT) pre-designed alt-R CRISPR Cas9 platform. Single-stranded DNA oligonucleotides (ssODNs) were purchased from IDT, with the *SORL1*-targeting gRNA sequence: CGACCAGGGTGAATAGGAAC.

CRISPR/Cas9 genome editing was performed as previously described (Bruntraeger et al., 2019). Briefly, Cas9/gRNA ribonucleoprotein complexes and ssODNs were transfected into iPSCs by electroporation using the Neon Transfection System according to the manufacturer’s instructions (Life Technologies). Cells were allowed to grow to 75% confluence, dissociated using Accutase and plated into Geltrex (Life Technologies)-coated 10 cm^2^ dish (Thermo Scientific) at a low density. Individual colonies were picked manually into 96 well plates for clonal expansion and *SORL1* KO clones were identified by Sanger sequencing.

#### Directed differentiation to human cortical neuron culture

Directed differentiation of iPSCs to cerebral cortex was carried out as previously described (Shi et al., 2012). Briefly, dissociated iPSCs were plated on 6-well plates coated with GelTrex (Life Technologies) and neural induction were initiated by changing into culture medium that supports neuronal differentiation and neurogenesis, a 1:1 mixture of N2- and B27-containing media (N2B27) (supplemented with 1μM dorsomorphin and 10μM SB431542 to inhibit TGFβ signaling during neural induction). Media was replaced every 24 hours. At day 12, neuroepithelial cells were harvested with dispase and replated in laminin-coated plates with FGF2-containing media for 4 days. FGF2 was then withdrew and dissociated using Accutase and neural progenitor cells were plated on GelTrex-coated plates. Plated neurons were maintained for up to 120 days with a medium change every 2-3 days.

#### RNA extraction and qRT-PCR analysis

Total RNA was extracted using RNeasy Mini Kit according to manufacturer protocol (QIAGEN). RNA was treated with DNase I (New England BioLabs) and 500 ng of RNA were retrotranscribed using the High-Capacity cDNA Reverse Transcription kit (Applied Biosystems). qRT-PCR were performed in a StepOnePlus instrument (Applied Biosystems) using the SYBR Green JumpStart Taq Ready Mix (Sigma) in a final volume of 15 μl, *APP* mRNA expression was assessed relative to GAPDH housekeeping gene using the specific primers reported in the Key Resources Table. Results were analyzed using the ABI StepOnePlus software (Thermo Fisher Scientific).

#### Protein analysis

Extracellular Aβ 1-42, Aβ 1-40, and Aβ 1-38 were measured in conditioned media using multiplexed MesoScale Discovery assays on a Quickplex SQ120 instrument (MesoScale Discovery) according to manufacturer instructions. Conditioned media from experiments collected at different time points were frozen at −80°C. Extracellular sAPPα and sAPPβ were measured in conditioned media using multiplexed MesoScale Discovery assays on a Quickplex SQ120 instrument (MesoScale Discovery).

#### Protein extraction and western blot analysis

For immunoblotting, whole cell lysate protein was extracted with RIPA buffer (Sigma) supplemented with protease inhibitors (Sigma) and Halt phosphatase inhibitors (ThermoFisher Scientific). Protein quantification was performed using Precision Red Advanced Protein Assay buffer (Cytoskeleton, Inc.). Samples were separated on a 4%–12% SDS-PAGE and transferred to PVDF membranes. Proteins were detected (Li-Cor Odyssey system) by incubation with specific primary antibodies and appropriate secondary antibodies. Antibodies used in this study are listed in Key Resources Table.

#### Confocal microscopy and image analysis

For live cell imaging, LAMP1-RFP (Catalogue no. C10597 from ThermoFisher Scientific), APP:VN and BACE1:VC were expressed in day 60-65 neurons. Neurons were washed and then replaced with fresh culture medium before imaging. Images were acquired using a Zeiss SP5 confocal microscope. Cultures were maintained at 37°C in a CO_2_ environment chamber.

For *in vitro* CTSD enzyme activity assay, neurons were incubated with BODIPY FL-Pepstatin A (Catalogue no. P12271 from Thermo Fisher Scientific) according to manufacturer instructions for 30 minutes at 37°C and imaged live using a Zeiss SP5 confocal microscope. Cultures were maintained at 37°C in a CO_2_ environment chamber.

For immunostaining, cells were fixed in 4% paraformaldehyde (PFA) in PBS followed by permeabilization with Triton X-100 (Sigma). Fixed cells were blocked with 10% normal goat serum (Sigma) in PBS, probed with primary antibodies diluted in blocking solution and detected with goat anti-mouse, anti-chicken or anti-rabbit secondary antibody coupled to Alexa Fluor 488 or 594. Confocal images were acquired using a Zeiss SP5 confocal microscope.

For the analysis of vesicle size, the image was first pre-processed to reduce noise using the subtract background command in ImageJ. The size of vesicles was then measured using the Particle Analysis command in ImageJ.

#### Antisense oligonucleotides

Antisense oligonucleotides (IDT) had the following modifications on a phosphorothioate (PS) backbone: a central gap region of ten 2′-deoxynucleotides flanked on both sides by five 2′-methoxyethyl nucleotides wings to improve nuclease resistance. All cytidine residues within the central gap region are 5-methyl deoxycytidine. The ASOs were labeled with 6-Carboxyfluorescein (FAM) at the 5′ end. The labeled ASOs were delivered to *SORL1*-null neurons by addition to extracellular media for a 10-day period. APP ASO sequence – TGTCACTTTCTTCAGCCAGT (Dobie, 2003).

### Quantification and statistical analysis

Unless otherwise specified, statistics analysis was performed using GraphPad Prism (Version 8). Student’s t test was used to compare differences between two groups. One-way ANOVA followed by post testing with Dunnett’s method was used to analysis differences between more than two groups. For precise p value calculation, a multiple t test was performed after ANOVA calculations. Significance threshold was defined as adjusted p value < 0.05. Data in Figure 4E are represented as box-and-whisker plots. Error bars in all figures represent SEM. The number of replicates (n) is listed in the legend of each figure.
